# Vascular endothelial growth factor-C expression and its relationship to pelvic lymph node status in invasive cervical cancer

**DOI:** 10.1054/bjoc.2001.1846

**Published:** 2001-07

**Authors:** I Hashimoto, J Kodama, N Seki, A Hongo, M Yoshinouchi, H Okuda, T Kudo

**Affiliations:** Department of Obstetrics and Gynecology and, Okayama University Medical School, 2-5-1 Shikata-cho, Okayama, 700-8558, Japan; Faculty of Health Sciences, Okayama University Medical School, 2-5-1 Shikata-cho, Okayama, 700-8558, Japan

**Keywords:** VEGF-C, lymph node metastasis, cervical cancer

## Abstract

Vascular endothelial growth factor-C (VEGF-C) has been implicated in lymphangiogenesis, the process of new lymphatics formation. The present study investigated VEGF-C mRNA expression in invasive cervical cancer tissue. Additionally, the association of VEGF-C mRNA with clinicopathological features was examined. VEGF-C mRNA expression was assessed by reverse transcription-polymerase chain reaction using β-action as an internal control. 75 patients presenting with invasive cervical cancer were included in the trial. VEGF-C mRNA expression was markedly higher in tumours in which pelvic lymph node metastasis was diagnosed by magnetic resonance (MR) imaging (*P* = 0.002). 53 patients displaying stage Ib–IIb cervical cancer underwent radical hysterectomy and pelvic lymphadenectomy. VEGF-C expression was significantly higher in tumours exhibiting deep stromal invasion, pelvic lymph node metastasis and lymph-vascular space involvement (*P* = 0.016, *P* = 0.006 and *P* = 0.036, respectively). Multivariate analysis revealed VEGF-C mRNA expression to be the sole independent factor influencing pelvic lymph node metastasis. Subjects demonstrating VEGF-C mRNA expression displayed significantly poorer prognoses than those lacking VEGF-C mRNA expression (*P* = 0.049). These findings provide evidence supporting the involvement of VEGF-C expression in the promotion of lymph node metastasis in cervical cancer. Furthermore, examination of VEGF-C expression in biopsy specimens may be beneficial in the prediction of pelvic lymph node metastasis. © 2001 Cancer Research Campaign http://www.bjcancer.com


*British Journal of Cancer* (2001) **85**(1), 93–97
© 2001 Cancer Research Campaign
doi: 10.1054/ bjoc.2001.1846, available online at http://www.idealibrary.com on IDEAL®

http://www.bjcancer.com

### Vascular endothelial growth factor-C expression and its relationship to pelvic lymph node status in invasive cervical cancer

**I Hashimoto[1], J Kodama[1], N Seki[1], A Hongo[1], M Yoshinouchi[1], H Okuda[2] and T Kudo[1]**

[1]Department of Obstetrics and Gynecology and [2]Faculty of Health Sciences, Okayama University Medical School, 2-5-1 Shikata-cho, Okayama 700-8558, Japan

**Summary** Vascular endothelial growth factor-C (VEGF-C) has been implicated in lymphangiogenesis, the process of new lymphatics formation. The present study investigated VEGF-C mRNA expression in invasive cervical cancer tissue. Additionally, the association of VEGF-C mRNA with clinicopathological features was examined. VEGF-C mRNA expression was assessed by reverse transcription-polymerase chain reaction using β-action as an internal control. 75 patients presenting with invasive cervical cancer were included in the trial. VEGF-C mRNA expression was markedly higher in tumours in which pelvic lymph node metastasis was diagnosed by magnetic resonance (MR) imaging ($P = 0.002$). 53 patients displaying stage Ib–IIb cervical cancer underwent radical hysterectomy and pelvic lymphadenectomy. VEGF-C expression was significantly higher in tumours exhibiting deep stromal invasion, pelvic lymph node metastasis and lymph-vascular space involvement ($P = 0.016$, $P = 0.006$ and $P = 0.036$, respectively). Multivariate analysis revealed VEGF-C mRNA expression to be the sole independent factor influencing pelvic lymph node metastasis. Subjects demonstrating VEGF-C mRNA expression displayed significantly poorer prognoses than those lacking VEGF-C mRNA expression ($P = 0.049$). These findings provide evidence supporting the involvement of VEGF-C expression in the promotion of lymph node metastasis in cervical cancer. Furthermore, examination of VEGF-C expression in biopsy specimens may be beneficial in the prediction of pelvic lymph node metastasis. © 2001 Cancer Research Campaign http://www.bjcancer.com

**Keywords**: VEGF-C; lymph node metastasis; cervical cancer

Due to the availability of cervical cytology screening programmes, the incidence of invasive cervical cancer is decreasing. Nevertheless, invasive cervical cancer remains a major health concern throughout the world. Disease stage is the most significant prognostic criterion in individuals presenting with invasive cervical cancer; however, patient survival following radical hysterectomy and pelvic lymphadenectomy is dependent on several factors. These factors include status of the lymph nodes, tumour size, paracervical involvement, depth of stromal invasion and lymph vascular space invasion (Lurain, 1996). The most highly dependent variable associated with survival is lymph node status. In fact, a favourable survival rate (5-year, disease-free survival, 87.9%) has been observed in patients displaying negative pelvic nodes, whereas a survival rate of 48.7% has been documented in patients demonstrating nodal involvement in our hospital (Kodama et al, 1999a). However, the mechanisms relating to lymph node metastasis are poorly examined.

It is well known that vascular endothelial growth factor (VEGF) plays an essential role in vasculogenesis and angiogenesis. In a previous study, we showed that VEGF expression is involved in the promotion of angiogenesis in cervical cancer (Kodama et al, 1999b). Several additional members of the VEGF family have been identified, including placenta growth factor (P1GF), VEGF-B, VEGF-C, VEGF-D and VEGF-E (Maglione et al, 1991; Kukk et al, 1996; Olofsson et al, 1996; Yamada et al, 1997; Meyer et al, 1999). VEGF-C binds to and induces autophosphorylation of the tyrosine kinase receptor VEGFR-3. VEGF-C also activates VEGFR-2 but not VEGFR-1 (Kukk et al, 1996). Other members of the VEGF family, with the exception of VEGF-D, are unable to activate VEGFR-3. VEGFR-3 is highly specific to the lymphatic endothelium in adult tissues (Kaipainen et al, 1995). Consequently, VEGF-C and VEGFR-3 have been implicated in lymphangiogenesis.

Recently, the correlation of VEGF-C expression in malignant tumours with lymph node metastasis has been reported (Bunono et al, 1999; Kurebayashi et al, 1999; Tsurusaki et al, 1999; Yonemura et al, 1999; Akagi et al, 2000). No investigations have been conducted regarding VEGF-C expression in invasive cervical cancer. The current study examined VEGF-C mRNA expression in invasive cervical cancer tissues as well as its association with clinicopathological features. A meaningful correlation between VEGF-C expression and pelvic lymph node metastasis was confirmed.

### MATERIALS AND METHODS

### Patients and tissue samples

The patient population for this study consisted of 75 individuals presenting with invasive cervical cancer (Department of Obstetrics and Gynecology of Okayama University Medical School, Okayama, Japan). Biopsy specimens were obtained prior to the initiation of treatment. Each specimen was partitioned into 2 equal portions. One sample was snap frozen and stored at –80˚C until

*Received 2 January 2001*
*Received 12 March 2001*
*Accepted 20 March 2001*

*Correspondence to:* J Kodama required for RNA extraction. The second sample was fixed in 10% formaldehyde solution for histopathologic examination. Specimens that did not display significant cancer cell numbers upon histopathological diagnosis were excluded from the study. Magnetic resonance (MR) imaging was performed on a 1.5-T superconducting magnet (Magnetom H-15; Siemens, Erlangen, Germany) in order to evaluate tumour size, local extension and lymph node metastasis.

Histologic cell types of the tumours were assigned according to the WHO classification. 40 cases were classified as squamous cell carcinoma, 22 as adenocarcinoma and 13 as adenosquamous carcinoma. Staging was reviewed based on the International Federation of Gynecology and Obstetrics (FIGO) staging system: 23, 38, 10 and 4 cases were stage Ib, stage II, stage III and stage IV, respectively. Median age at the time of treatment was 53 years (range 26–90 years). Radical hysterectomy and pelvic lymphadenectomy were performed in 53 subjects with stage Ib–IIb disease but otherwise exhibiting good physical condition. Patients displaying lymph node metastasis, parametrial involvement, deep stromal invasion or marked lymph–vascular space involvement were treated with adjuvant external whole pelvic irradiation (50 Gy) or adjuvant combination chemotherapy. The remaining 22 patients were treated primarily with radiotherapy or concurrent chemoradiotherapy. Disease-free survival was defined as the interval from initial therapy to the recurrence or to November 30, 2000. The median duration of follow-up was 28 months (range, 3–59). Disease recurred in 24 out of 75 subjects (32.0%). Additionally, 10 normal cervical specimens were obtained from patients demonstrating benign gynaecological disease.

### RNA preparation of sample

Total RNA was prepared from each specimen using an RNeasy Total RNA kit (QIAGEN, Hilden, Germany) according to the manufacturer's protocol. Tissues exhibiting RNA characterized by high quality 18S and 28S bands on ethidium bromide-stained gels were preferentially selected.

### Reverse transcriptase-polymerase chain reaction

RT was conducted according to the Thermoscript RT-PCR System (LIFE TECHNOLOGIES, Rockville, MD) protocol for reverse transcription of 3 ug total RNA in a total 20 ul reaction volume. Transcribed products were subjected to PCR for VEGF-C (sense primer; 5′-GAGGCTGGCAACATAACAGAG-3′, antisense primer; 5′-CCTTGAGAGAGAGGCACTGT-3′) and β-actin (sense primer; 5′-CTCACCATGGATGATGATAT-3′, antisense primer; 5′-TGG-GTCATCTTCTCGCGGTT-3′) (Fujita et al, 1994; André et al, 2000). VEGF-C cDNA amplification was initiated with denaturation for 3 min at 94˚C followed by 30 cycles of 30-s denaturation at 94˚C. Annealing was then effected at 59˚C for 1 min followed by a 1 min extension at 72˚C. The PCR profile for β-actin consisted of an initial denaturation of 3 min at 94˚C followed by 30 cycles of 1-min denaturation at 94˚C, 1-min annealing at 55˚C and a 1-min extension at 72˚C. The PCR mixture was maintained at 72˚C for 15 min for final extension. The details of PCR reaction mixture have been described elsewhere (Seki et al, 1997). Final PCR products were subsequently electrophoresed on a 2% agarose gel and stained with ethidium bromide. UV-illuminated gels were photographed using Polaroid Type 667 films. Photographs were quantitated with an image scanner GT-9500 (EPSON, Suwa, Japan) and analysed with Basic Quantifier software (Bio Image, Ann Arban, MI).

cDNA amounts were corrected by β-actin as an internal standard in order to obtain semi-quantification of VEGF-C mRNA levels. For this reason, a technique based on a competitive PCR approach involving a nonhomologous internal standard was employed (COMPETITOR; Competitive DNA Construction Kit; TAKARA, Kyoto, Japan). 1 ul cDNAs derived from samples were co-amplified in the presence of serial dilutions of β-action COMPETITOR. The point of equal intensity between the bands of β-actin COMPETITOR and the cDNA template was evaluated. cDNAs in the presence of $1 \times 10^5$ copies of β-actin were subsequently utilized in the amplification of VEGF-C gene. PCR products derived from VEGF-C gene were assigned to the positive (+) or negative (–) VEGF-C expressing groups.

### Immunohistochemical staining for microvessels

Expression of factor VIII-related antigen was assessed in 53 formalin-fixed, paraffin-embedded sections obtained at the time of radical surgery by the avidin-biotin complex (ABC) procedure. Anti-factor VIII-related monoclonal antibody (DAKOPATTS, Copenhagen, Denmark) was utilized as a primary antibody. The entire tumorous lesion was scanned under low-power magnification in order to select regions displaying the most intense vascularization. The number of microvessels was recorded by counting any positively stained endothelial cell or endothelial cell cluster as a single, countable microvessel in a 100 × microscopic field. In most cases, the microvessel density was higher at the invasive edge of the tumour than within the tumour. The 10 most neovascularized regions were selected as a minimum. The mean of the top 3 counts was used as the microvessel count for each case. Microvessel number was determined by an investigator who had no knowledge of the VEGF-C expression.

### Statistical analyses

Association between the variables were tested using the Chi-square test, Fisher's exact probability test, Mann–Whitney *U*-test or stepwise logistic regression analysis. Survival rates were calculated by the Kaplan–Meier method and differences were examined by the log-rank test. Factors found to be significant were then chosen for stepwise Cox's multivariate proportional hazard model in order to ascertain their prognostic values. These analyses were performed utilizing the Stat-View 5.0 software (Abacus Concepts, Berkeley, CA). Probability values less than 0.05 were considered statistically significant.

### RESULTS

### VEGF-C mRNA expression in normal cervix and cervical cancer

Figure 1 displays representative photographs of the final RT-PCR products for VEGF-C in normal cervix (cases 1–3) and invasive cervical cancer (cases 4–10). VEGF-C mRNA expression was detected in only 1 of 10 normal cervical specimens (10.0%). In contrast, VEGF-C mRNA expression was detected in 36 of 75 invasive cervical cancer samples (48.0%). The frequency of detectable VEGF-C mRNA of invasive cervical cancer was markedly higher than that of normal cervix ($P = 0.017$).

*© 2001 Cancer Research Campaign*

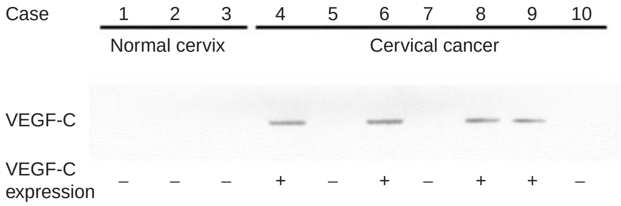

**Figure 1** Detection of mRNA for VEGF-C in normal cervix and invasive cervical cancer. Total RNAs from biopsy specimens were extracted, transcribed to cDNAs and subjected to PCR for VEGF-C. Cases 1–3; normal cervix, cases 4–10; invasive cervical cancer

**Table 1** Association between VEGF-C mRNA expression and clinicopathological factors in cevical cancer

| | No. of cases | VEGF-C | | *P* value |
|---|---|---|---|---|
| | | (+) | (–) | |
| Age | | | | |
| <50 | 37 | 22 | 15 | NS |
| ≥50 | 38 | 14 | 24 | |
| Histological cell type | | | | |
| SCC | 53 | 28 | 25 | NS |
| ADE | 22 | 8 | 14 | |
| Stage | | | | |
| I+II | 61 | 29 | 32 | NS |
| III+IV | 14 | 7 | 7 | |
| Pelvic lymph node status[a] | | | | |
| negative | 61 | 24 | 37 | *P* = 0.002 |
| positive | 14 | 12 | 2 | |

SCC, squamous cell carcinoma; ADE, adenocarcinoma; [a] pelvic lymph node status assessed by MR imaging; NS, not significant.

The frequency of detectable VEGF-C mRNA was significantly higher in tumours exhibiting pelvic lymph node metastasis assessed by MR imaging ($P = 0.002$) (Table 1). No meaningful differences in VEGF-C mRNA expression with respect to age, histological cell type and clinical stage were observed.

### VEGF-C mRNA expression and clinicopathological features in cervical cancer treated with radical hysterectomy and pelvic lymphadenectomy

The frequency of detectable VEGF-C mRNA was significantly higher in tumours exhibiting deep stromal invasion, pelvic lymph node metastasis and lymph–vascular space involvement ($P = 0.016$, $P = 0.006$ and $P = 0.036$, respectively) (Table 2). There was no correlation between VEGF-C mRNA expression and other clinicopathological factors (Table 2). The mean microvessel counts in tumours exhibiting and lacking VEGF-C mRNA expression were 37.5 ± 4.8 and 37.4 ± 5.3, respectively (not significant).

Pelvic lymph node metastasis was correlated with tumour size, stromal invasion, parametrial invasion, lymph–vascular space involvement and VEGF-C mRNA expression. Multivariate analysis revealed VEGF-C mRNA expression to be the exclusive independent factor influencing pelvic lymph node metastasis (Table 3). As a result, VEGF-C mRNA expression was further compared with MR imaging in the evaluation of pelvic lymph node metastasis. Sensitivity, specificity, positive and negative predictive values and accuracy of VEGF-C mRNA expression and MR imaging in the diagnosis of pelvic lymph node metastasis were 81.3%, 62.2%, 48.1%, 88.5%, 67.9% and 43.8%, 100%, 100%, 80.4%, 83.0%, respectively.

**Table 2** Association between VEGF-C mRNA expression and clinicopathological factors in cervical cancer treated with radical hysterectomy and pelvic lymphadenectomy

| Variables | No. of cases | VEGF-C | | *P* value |
|---|---|---|---|---|
| | | (+) | (–) | |
| Age | | | | NS |
| < 50 | 34 | 19 | 15 | |
| ≥ 50 | 19 | 8 | 11 | |
| Histological cell type | | | | NS |
| SCC | 34 | 20 | 14 | |
| ADE | 19 | 7 | 12 | |
| Tumour size (cm) | | | | NS |
| ≤ 4 | 38 | 19 | 19 | |
| > 4 | 15 | 8 | 7 | |
| Stromal invasion | | | | *P* = 0.016 |
| ≤ 2/3 | 20 | 6 | 14 | |
| > 2/3 | 33 | 21 | 12 | |
| Parametrial invasion | | | | NS |
| Negative | 33 | 14 | 19 | |
| Positive | 20 | 13 | 7 | |
| Vaginal invasion | | | | NS |
| Negative | 44 | 22 | 22 | |
| Positive | 9 | 5 | 4 | |
| Lymphnode metastasis | | | | *P* = 0.006 |
| Negative | 37 | 14 | 23 | |
| Positive | 16 | 13 | 3 | |
| LVS involvement | | | | *P* = 0.036 |
| Negative | 21 | 7 | 14 | |
| Positive | 32 | 20 | 12 | |

SCC, squamous cell carcinoma; ADE, adenocarcinoma; LVS, lymph-vascular space; NS, not significant.

### Association of VEGF-C mRNA expression with survival

Figure 2A presents the disease-free survival curves of patients in 75 patients displaying invasive cervical cancer according to the VEGF-C mRNA expression status. A tendency between VEGF-C mRNA expression and poorer outcome was noted; however, the relationship was not statistically significant ($P = 0.068$). Figure 2B shows the disease-free survival curves in 53 instances of invasive cervical cancer treated with radical hysterectomy and pelvic lymphadenectomy. Patients exhibiting VEGF-C mRNA expression demonstrated a markedly poorer prognosis than those lacking VEGF-C mRNA expression ($P = 0.049$). Deep stromal invasion, parametrial invasion, lymph–vascular space involvement, pelvic lymph node metastasis and high microvessel count were also found to be significant in disease-free survival. Microvessel count was an only independent prognostic factor in the multivariate proportional hazard model.

### DISCUSSION

In the present study, VEGF-C mRNA expression in normal cervix and invasive cervical cancer specimens was investigated. We showed that the frequency of detectable VEGF-C mRNA of invasive cervical cancer is significantly higher than that of normal cervix. This finding is consistent with other reports, which demonstrated elevated VEGF-C expression in tumour tissues in contrast to the low levels observed in the normal tissues (Bunone et al, 1999; Ohta et al, 1999; Valtola et al, 1999; Yonemura et al, 1999; Akagi et al, 2000). Consequently, we hypothesize that VEGF-C

© 2001 Cancer Research Campaign

**Table 3** Univariate and multivariate analysis with respect to lymph nodes metastasis

| | | Univariate | | | Multivariate | | |
|---|---|---|---|---|---|---|---|
| **Variable** | **Comparison** | **Odds ratio** | ***P* value** | **95% confidence interval** | **Odds ratio** | ***P* value** | **95% confidence interval** |
| Histological cell type | SCC : ADE | – | NS | | | | |
| Tumour size (cm) | ≤4 : >4 | 4.3 | $P = 0.026$ | 1.192–15.409 | – | NS | |
| Stromal invasion | ≤ 2/3 : > 2/3 | 15.8 | $P = 0.011$ | 1.892–132.521 | – | NS | |
| Parametrial invasion | Negative : Positive | 4.5 | $P = 0.018$ | 1.295–15.635 | – | NS | |
| Vaginal invasion | Negative : Positive | – | NS | | | | |
| LVS involvement | Negative : Positive | 7.4 | $P = 0.015$ | 1.468–37.185 | – | NS | |
| VEGF-C mRNA | Negative : Positive | 7.1 | $P = 0.007$ | 1.720–29.467 | 6.7 | $P = 0.029$ | 1.208–35.582 |

SCC, squamous cell carcinoma; ADE, adenocarcinoma; LVS, lymph-vascular space: NS, not significant.

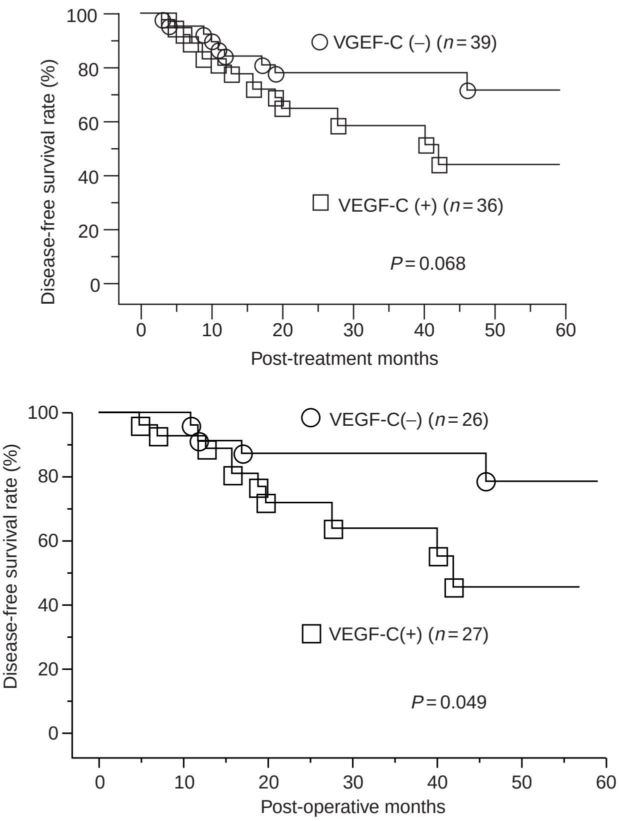

**Figure 2 (A)** Disease-free survival curves of 75 patients with invasive cervical cancer according to VEGF-C mRNA expression. (**B**) Disease-free survival curves of 53 patients with stage Ib–IIb cervical cancer underwent radical hysterectomy and pelvic lymphadenectomy according to VEGF-C mRNA expression may contribute to the metastatic process, carrying cancer cells into the lymphatic vessels.

VEGF-C mRNA expression was further examined so as to determine correlation with clinicopathological features in invasive cervical cancer. The current results clearly demonstrated that the frequency of detectable VEGF-C mRNA was markedly increased in cases where lymph–vascular space involvement and pelvic lymph node metastasis were in evidence. The present data are compatible with reports that positive correlation between VEGF-C expression and lymphatic invasion was observed in cases of colon cancer, gastric cancer and lung adenocarcinoma (Yonemura et al, 1999; Akagi et al, 2000; Niki et al, 2000). A meaningful correlation between VEGF-C expression and lymph node metastasis has been reported in cases of colon cancer, prostatic carcinoma, breast cancer, gastric cancer and thyroid cancer (Bunono et al, 1999; Kurebayashi et al, 1999; Tsurusaki et al, 1999; Yonemura et al, 1999; Akagi et al, 2000). These findings indicate that VEGF-C expression is implicated in lymphatic invasion and lymph node metastasis. Although lymphatic vessel counts were not measured in this study, several investigations showed a close correlation between VEGF-C and VEGFR-3 expression (Tsurusaki et al, 1999; Gunningham et al, 2000; Yonemura, 1999). We postulate that VEGF-C stimulates lymphangiogenesis and enhances the invasion of cancer cells via loosing of lymphatic endothelial cells, resulting in lymph node metastasis.

Lymphangiography (LAG), computed tomography (CT) or magnetic resonance (MR) imaging were employed in the diagnosis of lymph node metastasis in patients presenting with invasive cervical cancer. The LAG, CT and MR imaging provide similar performance in the detection of lymph node metastasis derived from cervical cancer (Scheidler et al, 1997). MR imaging has been commonly utilized in the evaluation of invasive cervical cancer in our hospital. Interestingly, multivariate analysis revealed that VEGF-C mRNA expression in biopsy specimens is the sole independent factor influencing pelvic lymph node metastasis in the present study. Therefore, MR imaging was compared with VEGF-C mRNA expression in the evaluation of pelvic lymph node metastasis. A trend toward higher accuracy was displayed by MR imaging relative to that for VEGF-C mRNA expression. However, it is noteworthy that VEGF-C mRNA expression offered markedly higher sensitivity in the detection of pelvic lymph node metastasis.

Determination of the identity of angiogenic factors involved in the mediation of angiogenesis is of paramount importance. Consequently, this information could afford novel methods for therapeutic intervention in this disease. In a previous study, we showed that the expression of VEGF-A is involved in the promotion of angiogenesis in cervical cancer (Kodama et al, 1999b). VEGF-C is a ligand for VEGFR-2; therefore, we examined the correlation between VEGF-C mRNA expression and vascularization in cervical cancer. Our study demonstrated that VEGF-C gene expression is not correlated with tumour vascularity in invasive cervical cancer. In accordance with our report, VEGF-C gene or protein expression is reported not to be correlated with microvessel count in colorectal cancer, mesothelioma, prostatic carcinoma and breast cancer (Ohta et al, 1999; Tsurusaki et al,

*© 2001 Cancer Research Campaign*

1999; Akagi et al, 2000; Gunningham et al, 2000). Accordingly, VEGFR-2 may not be a primary receptor of VEGF-C in these tumours. In fact, VEGF-C displays greater affinity for VEGFR-3 than for VEGFR-2. Furthermore, proteolytic processing of VEGF-C generates several VEGF-C forms possessing increased activity toward VEGFR-3; however, the fully processed VEGF-C can activate VEGFR-2 (Joukov et al, 1997).

The current study also indicated that patients with VEGF-C mRNA expression exhibit poorer prognoses than those lacking VEGF-C mRNA expression. VEGF-C, which is strongly correlated to pelvic lymph node metastasis, may influence prognosis. However, VEGF-C mRNA expression was not an independent prognostic factor among our study population. Consequently, VEGF-C expression dose not appear to be a useful prognostic factor in cases of cervical cancer. VEGF-D, which is 48% identical with VEGF-C, may also bind to and activate VEGFR-3 (Yamada et al, 1997). Consequently, VEGF-D might play a crucial role in lymph node metastasis in addition to influencing prognosis. Further investigation is necessary in order to clarify this issue.

In conclusion, our findings indicate that VEGF-C expression is involved in the promotion of lymph node metastasis in cervical cancer. Furthermore, examination of VEGF-C expression in biopsy specimens may be useful as a predictor of pelvic lymph node metastasis.

### REFERENCES

Akagi K, Ikeda Y, Miyazaki M, Abe T, Kinoshita J, Maehara Y and Sugimachi K (2000) Vascular endothelial growth factor-C (VEGF-C) expression in human colorectal cancer tissues. *Brit J Cancer* **83**: 887–891

André T, Kotelevets L, Vaillant JC, Coudray AM, Weber L, Prévot S, Parc R, Gespach C and Chastre E (2000) VEGF, VEGF-B, VEGF-C, and their receptors KDR, Flt-1 and flt-4 during the neoplastic progression of human colonic mucosa. *Int J Cancer* **86**: 174–181

Bunone G, Vigneri P, Mariani L, Butó S, Collini P, Pilotti S, Pierotti MA and Bongarzone I (1999) Expression of angiogenesis stimulators and inhibitors in human thyroid tumors and correlation with clinical pathological features. *Am J Pathol* **155**: 1967–1976

Fujita N, Yaegashi N, Ide Y, Sato S, Nakamura M, Ishiwata I and Yajima A (1994) Expression of CD44 in normal human versus tumor endometrial tissues: possible implication of reduced expression of CD44 in lymph-vascular space involvement of cancer cells. *Cancer Res* **54**: 3922–3928

Gunningham SP, Currie MJ, Han C, Robinson BA, Scott PAE, Harris AL and Fox SB (2000) The short form of the alternatively spliced flt-4 but not its ligand vascular endothelial growth factor C is related to lymph node metastasis in human breast cancers. *Clin Cancer Res* **6**: 4278–4286

Joukov V, Sorsa T, Kumar V, Jeltsch M, Claesson-Welsh L, Cao Y, Saksela O, Kalkkinen N and Alitalo K (1997) Proteolytic processing regulates receptor specificity and activity of VEGF-C. *EMBO J* **16**: 3898–3911

Kaipainen A, Korhonen J, Mustonen T, van Hinsbergh VWM, Fang G-H, Dumont D, Beitman M and Alitalo K (1995) Expression of the fms-like tyrosine kinase 4 gene becomes restricted to lymphatic endothelium during development. *Proc Natl Acad Sci USA* **92**: 3566–3570

Kodama J, Yoshinouchi M, Seki N, Hongo A, Miyagi Y and Kudo (1999a) Angiogenesis and platelet-derived endothelial cell growth factor/thymidine phosphorylase expression in cervical cancer. *Int J Oncol* **15**: 149–154

Kodama J, Seki N, Tokumo A, Hongo A, Miyagi Y, Yoshinouchi M, Okuda H and Kudo T (1999b) Vascular endothelial growth factor is implicated in early invasion of cervical cancer. *Eur J Cancer* **35**: 485–489

Kukk E, Lymboussaki A, Taira S, Kaipanen A, Jeltsch M, Joukov V and Alitalo K (1996) VEGF-C receptor binding, and pattern of expression with VEGFR-3 suggests a role in lymphatic vascular development. *Development* **122**: 3829–3837

Kurebayashi J, Otsuki T, Kunisue H, Mikami Y, Tanaka K, Yamamoto S and Sonoo H (1999) Expression of vascular endothelial growth factor (VEGF) family members in breast cancer. *Jpn J Cancer Res* **90**: 977–981

Lurain JR (1996) Uterine cancer. In: *Novaks Gynecology*, Berek JS (ed) pp 1057–1110. Williams and Wilkins: Maryland

Maglione D, Guerriero V, Viglietto G, Delli-Bovi P and Persico MG (1991) Isolation of a human placental cDNA coding for a protein related to the vascular permeability factor. *Proc Natl Acad Sci USA* **88**: 9267–9271

Meyer M, Clauss M, Lepple-Wienhues A, Waltenberger J, Augustin HG, Ziche M, Lanz C, Buttner M, Rziha HJ and Dehio C (1999) A novel vascular endothelial growth factor encoded by Orf virus, VEGF-E, mediates angiogenesis via signalling through VEGFR-2 (KDR) but not VEGFR-1 (Flt-1) receptor tyrosine kinases. *EMBO J* **18**: 363–374

Niki T, Iba S, Tokunou M, Yamada T, Matsuno Y and Hirohashi S (2000) Expression of vascular endothelial growth factor A, B, C, and D and their relationships to lymph node status in lung adenocarcinoma. *Clin Cancer Res* **6**: 2431–2439

Ohta Y, Shridhar V, Bright RK, Kalemkerian GP, Du W, Carbone M, Watanabe Y and Pass HI (1999) VEGF and VEGF type C play an important role in angiogenesis and lymphangiogenesis in human malignant mesothelioma tumours. *Brit J Cancer* **81**: 54–61

Olofsson B, Pajusola K, Kaipanen A, von Euler G, Joukov V, Saksela O, Orpana A, petterson RF, Alitalo K and Eriksson U (1996) Vascular endothelial growth factor B, a novel growth factor for endothelial cells. *Proc Natl Acad Sci USA* **93**: 2576–2581

Scheidler J, Hricak H, Yu KK, subak L and Segal MR (1997) Radiological evaluation of lymph node metastases in patients with cervical cancer. A mete-analysis. *JAMA* **278**: 1096–1101

Seki A, Kodama J, Miyagi Y, Kamimura S, Yoshinouchi M and Kudo T (1997) Amplification of the mdm-2 gene and p53 abnormalities in uterine sarcomas. *Int J Cancer* **73**: 33–37

Tsurusaki T, Kanda S, Sasaki H, Kanetake H, Saito Y, Alitalo K and Koji T (1999) Vascular endothelial growth factor-C expression in human prostatic carcinoma and its relationship to lymph node metastasis. *Brit J Cancer* **80**: 309–313

Valtola R, Salven P, Heikkilä P, Taipale J, Joensuu H, Rehn M, Pihlajaniemi T, Weich H, de Waal R and Alitalo K (1999) VEGFR-3 and its ligand VEGF-Care associated with angiogenesis in breast cancer. *Am J Pathol* **154**: 1381–1390

Yamada Y, Nezu J, Shimane M and Hirata Y (1997) Molecular cloning of a novel vascular endothelial growth factor, VEGF-D. *Genomics* **42**: 483–488

Yonemura Y, Endo Y, Fujita H, Fushida S, Ninomiya I, Bandou E, Taniguchi K, Miwa K, Ohoyama S, Sugiyama K and Sasaki T (1999) Role of vascular endothelial growth factor C expression in the development of lymph node metastasis in gastric cancer. *Clin Cancer Res* **5**: 1823–1829

© 2001 Cancer Research Campaign
